# Measurements of the Vertical Displacements of a Railway Bridge Using TLS Technology in the Context of the Upgrade of the Polish Railway Transport

**DOI:** 10.3390/s19194275

**Published:** 2019-10-02

**Authors:** Pelagia Gawronek, Maria Makuch, Bartosz Mitka, Tadeusz Gargula

**Affiliations:** 1 Department of Land Surveying, Faculty of Environmental Engineering and Land Surveying, University of Agriculture in Krakow, Balicka 253a Street, 30-149 Krakow, Poland; m.makuch@ur.krakow.pl (M.M.); t.gargula@ur.krakow.pl (T.G.); 2 Department of Agricultural Land Surveying, Cadaster and Photogrammetry, Faculty of Environmental Engineering and Land Surveying, University of Agriculture in Krakow, Balicka 253a Street, 30-149 Krakow, Poland; b.mitka@ur.krakow.pl

**Keywords:** displacement and deformation, surveying engineering, period measurements, 3D technology measurement, terrestrial laser scanning, point cloud analysis, registration, filtration, differential model, Polish railway bridges

## Abstract

The railway system in Poland is undergoing technological transformation. The development of the Polish railway system concerns not only high-speed trains but also infrastructure. The steel bridge is the most popular type of railway bridge in Poland. Most of them were built in the 1950s and 1960s. According to the recommendations in place in Western Europe, such railway bridges should be reviewed in terms of their fitness for use with modern high-speed trains. The modern technological revolution affects not only the railway, but also developments in displacement and deformation measurement techniques. New technologies provide more objective measurement results and accelerate results processing. They also facilitate the non-contact measurement of bridge structure stability. The authors investigated the vertical displacement of an old steel railway bridge in three different, specific case studies of terrestrial laser scanning data application. Then, the results of 3D data were compared with traditional land surveying results. The scientific results led to a conclusion that a strictly determined methodology of the measurement and analysis of a terrestrial laser scanner results supported by traditional land surveying techniques facilitates the determination of the vertical displacement of bridges with acceptable accuracy.

## 1. Introduction

Bridge infrastructure is a foundation of the land transport system. Its importance for road engineering poses strict requirements for reliable and fault-free use. The matter of trouble-free maintenance of bridges is an important issue not only from the point of view of the functionality of the object, which affects the economy directly, but also for financial reasons related to high costs of construction, upgrading, and potential decommissioning of the structures. Apart from the economic and financial factors, good repair of bridges contributes to the preservation of the regional historical and landscape heritage. The diversity of causes of the correct use often results in the “stimulation (extension) of the service life” of bridges [1]. Therefore, the ground rule for sustainable operation should be periodic diagnostics of stability.

Research on the condition of railway infrastructure and its relation to the rolling stock movement, which was initiated in the mid-19th century (after the disaster of the Dee River railway bridge in Chester) provided the basis for formulating the first technical standards for the safety of these facilities [2]. In the 1950s, the service speed of rolling stocks did not exceed 100 km·h^−1^. Today, the freight rolling stock reaches speeds of 160 km·h^−1^, while passenger rolling stock reaches speeds of even up to 350 km·h^−1^ [3]. The technological potential of railway rolling stock, which results from the European trend to develop high-speed rail, must be harmonized with the technical possibilities of railway infrastructure (often age-old). It seems logical that the standards and procedures for testing the stability of railway civil engineering structures, which include new measurement technologies, should be continuously updated.

**Figure 1:**
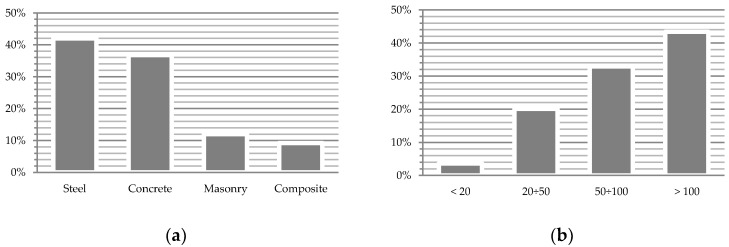
Statistics of railway bridge infrastructure; (**a**) according to construction material specifications; (**b**) according to the number of years in operation (based on [1]).

Currently, a large part of the railway bridge infrastructure in Poland has reached the upper limit of its design life (Figure 1). These objects, which were usually designed for service loads smaller than the actual operating loads today, are subject to accelerated structural degradation as a consequence of material fatigue [4]. The service life of steel girders of railway bridges is assumed by PKP PLK S.A. (the Polish national railway) not to exceed 100 years of operation [5]. Currently, almost half of Polish railway bridges have reached an advanced age past the durability period defined in technical projects [1]. Bień describes the existing state as “pathology and technical geriatrics of Polish railway bridges” [1].

The poor technical condition of the railway transport in Poland regrettably contributes to the poor rating of the railway system performance. The 2015 European Railway European Performance Index (RPI), which covers a combination of intensity of use, quality of service, and safety issues in a country’s railway sector, ranks PKP PLK S.A. 23 among 25 European states in the group of countries with poor performance results (tier 3). The 2015 RPI does not only put Poland at the end of the European rail performance spectrum, but also testifies about still developing economies [6,7]. However, the railway system performance assessment indicates that the poor quality of service does not result in a decrease in the intensity of use, which is very good. The popularity of the railway transport in Poland despite its poor technical condition and service quality may be a decisive factor contributing to its significant share in the transport market in the future. However, in order to achieve this goal, the upgrade process of the Polish railway, which has been in place for less than 10 years, has to be effective and prudent regarding the reconstruction or construction of infrastructure, and set on new technologies. The successful completion of the upgrade by PKP PLK S.A. in 2023, which will cost about EUR 16 billion (EU funding accounts for 60% of the total sum), and its transformation into high-speed rail will depend largely on the selective construction, upgrade, and restoration of railway infrastructure. 

According to the recommendations of the Polish government [8], the formal basis for conducting stability tests of railway bridges, which includes the technical condition and load-bearing capacity of the structure, is set in PKN standards (Polish Committee for Standardization), UIC regulations (Union Internationale des Chemins de fer) [9], and PKP PLK S.A.’s regulations. The internal regulations of PKP PLK S.A. [10,11,12] and PKN standards [13,14,15] concern mainly the acceptance testing of railway bridges. They further give research units a considerable level of freedom as regards the standards and procedures for the periodic diagnostics of railway bridges in Poland. According to them, the main recommendation for determining the stability of railway bridges is the one included in the design documentation. Unfortunately, design documentation for old objects very often consists solely of a building permit design. Also, the PKN standards and PKP PLK S.A.’s regulations are inconsistent in terms of static load tests and dynamic load tests (Table 1). The fundamental problem is to determine the objects that are formally required to undergo the tests. More inconsistencies in the regulations can be found for methods of conducting load tests, forcing vibrations, speeds used, measured parameters, determined parameters, and the interpretation of results [10,11,12,14,15].

There are no generally accepted test procedures, especially for railway bridge static and dynamic load tests by research units [16]. The lack of test standardization allows research units considerable freedom, which results in issues when comparing periodic tests and consequently prevents comparative analyses in the future. The necessity to standardize the standards and regulations regarding the stability testing of railway bridges is real. Under the current conditions, the PKN standards in the field of displacement and deformation measurements are complementary legislation. They recommend assessing the safety of structures with remote measurement methods and recommend new methods of performing stability tests, provided that they are scientifically justified and will be carried out by scientific and research institutions [14,17].

Terrestrial laser scanner (TLS) is an active remote sensing system that determines the coordinates of points (X, Y, Z) of a specific object based on an electronic measurement of the distance and angle. The measurement accuracy of terrestrial laser scanners determines the quality of TLS data. It is defined as the accuracy of the detail mapping of the object, the density of the captured point cloud of the object, and the degree of noise reduction. The final quality of TLS data is a consequence of the instrument parameters, e.g., measurement resolution [18], laser beam divergence, laser beam diameter [19,20], geometrical model of the scanner axes [21]; as well as measurements conditions, e.g., the physical features of the measured object [22], the location of the scanner relative to the measured object [23], the weather [20], and the methods of capturing and processing TLS data [24]. 

The stability investigation of objects using terrestrial laser scanning usually consists of the verification of whether a huge amount of point cloud data can compensate for their lower accuracy and provide the basis to determine their displacement and deformation [25]. Stability surveys of civil engineering structures using TLS can be classified into three different approaches. The first approach is to estimate the differential models from point clouds that were captured during consecutive periodic measurements. Algorithms of comparing two point clouds are available in most open access software. This point-to-point approach also has some disadvantages, e.g., limited sensitivity to determining small deformations [26,27]. For this reason, it is necessary to capture very high-density point clouds. This way, it is possible to determine relatively small displacements or deformations. It also facilitates checking for any systematic errors [19]. The point-to-point approach to detecting changes in bridge structures was applied by Zogg and Ingensand [28] and Shen-En [29]. These studies underlined the millimeter accuracy displacement determination and the simplicity of the approach. Considering that terrestrial laser scanners measure the same objects but never measure the same points during periodic measurements, the differential analysis should be carried out for generalizations of point clouds–surface meshes rather than for point clouds [30]. This approach necessitates the use of commercial software to generate surface meshes. The surface-to-surface approach was usually used in the monitoring of landslides [31] or determining the erosive progress of rock cliffs [32] in recent years. In addition to determining the natural effects of degradation, the methods have been used to determine the displacements of monolithic objects such as water dams [33] or cooling towers [34]. Today, this approach is increasingly popular in stability surveys of civil engineering structures such as bridges [35]. The second approach to the application of the TLS technology in the investigation of the stability of civil engineering objects, which can be described by geometric equation, is to fit point clouds into geometrical models, e.g., semi-cylinders (tunnel) [25], cylinders (chimney) [36], planes (e.g., pavement of bridges) [37], or other kinds of cuboids. In this approach, the TLS data of objects with regular geometry provides a comprehensive assessment of their imperfections, and therefore outclasses the redundant method of reflectorless tacheometry. The third approach is the analysis of changes in the structure over time based on longitudinal and transverse cross-sections from point clouds. The cross-sections approach is particularly useful in studies on underground objects, i.e., tunnels, caves, and grottos [38], or mining excavation [39]. The relevance of this approach was confirmed by studies on the stability of a Danube River bridge during static load tests [40] or regularity tests of the construction of the tunnel under the airport in Zaventem [41].

The authors of the article, inspired by industry needs and the capabilities of TLS technologies, assessed the applicability of terrestrial laser scanning in determining the displacement of a bridge in static conditions. The research was aimed at developing the most technically and economically justified methodology of capturing, processing, and managing TLS data. For this purpose, the vertical displacements of a bridge were determined in three different, specific case studies for TLS data. Their results were compared with the ones from traditional land surveying techniques. A strictly defined methodology of measuring and processing TLS data in synergy with traditional land surveying measurements was assumed to facilitate a reliable determination of the epochal vertical displacements of whole bridges. The proposed methodology for conducting measurements and processing object displacement data has been verified by the authors for an individual bridge span and during static load tests. During measurements, the same measuring stations were used both before and after static load tests (without changing the position of the tripods). In addition, only two scanning stations were registered with each other. It was enough to get 3D data of an individual bridge span. The results of the studies indicated that it is possible to determine the vertical displacement of a loaded object at ±1.0 mm accuracy [42]. This paper presents a broader study. It consists of epoch measurements of the whole object over almost three years. The measurements required not only scanning, but also epoch surveys of control network stability. For the purposes of this article, not only the object was scanned from two invariant stations before and after static load tests (as in studies [42]), but point clouds of the entire object were also captured. Moreover, before each epochal scanning, the control network was researched by stability tests. It was a necessity to be able to use the coordinate of the control network during each epoch registration. In contrast to the use of TLS data during static load tests [42], epoch research focused on the need and possible consequences of using a reliable control network. Determining the object changes by point clouds obtained from the same, unchanging scanner positions [42] may not give the same results as epochal scanning. Epochal scanning must take into account measurement errors (leveling, centering) as well as changes in the control network. As a consequence, vertical displacements from epochal TLS data can be more unreliable than those obtained during the static load test [42].

## 2. Materials and Methods

The endeavor to determine the methodology for the stability testing of bridges using TLS necessitated epochal measurements of an object that was in a static condition during the measurements. In order to minimize the influence of factors differentiating the measurement results, the following assumptions were made:

- the research object was specifically defined,
- the same variants of object monitoring were set during subsequent periodic measurements,
- measurement methods and measurement technologies were set that would be sufficiently accurate, and
- a research schedule was set to ensure timely periodic measurements (according to [43]).

### 2.1. Object

The empirical research was carried out on a bridge at kilometer (km) 2.485 of railway line No. 098 Sucha Beskidzka—Chabówka. The railway bridge represents a population of steel railway bridges that were built in the 1960s. The bridge is a two-span, continuous superstructure with a design span between centers of supports of 52.700 m. The structure of the railway bridge is supported by concrete, inclined abutments and a support. The total width of the railway bridge is 5.400 m. The object has a single track, 4.500 m gauge. The maximum total acceptable load of the railway bridge is 6.980 kg·cm^−2^. The theoretical deflection of the bridge, caused by the weight of the object, is 3.700 cm; the deflection caused by the dynamic load is 1.820 cm, and the maximum horizontal movement of the bridge equals ±3.200 cm.

For many years of use, the diagnostics of the railway bridge was limited to periodic inspections that assessed the overall condition of the structure. The theoretical static parameters of the more than 50-year-old object remain valid, even though there was a revolution in rail transport in recent decades, which redefined the conditions for the operation of the infrastructure. According to reports ORE D154/R4 (1985) and ERRI D 181/DT 329 (1995), railway bridge infrastructure should be re-evaluated in terms of theoretical static parameters due to the operational fatigue of the structure and new technical transport conditions. The review should possibly lead to an upgrade plan [3]. 

### 2.2. The Concept of Object Measurement

The concept of the object measurement assumes three variants of the measurement of the bridge: precise leveling of bridge spans (I), reflectorless precision tacheometry of controlled points (II), and terrestrial laser scanning (III) (Table 2). 

Each of the variants led to the determination of periodic vertical displacements of the object. Also, variants (**I**) and (**II**) were reference measurements for the TLS results. Each variant of determining the stability of the railway bridge was used in every measuring epoch simultaneously. Thus, they represented the same state of construction. Variants (**II**) and (**III**) required additional measurements for reference purposes. Therefore, the applied measurement methods were used directly to determine the stability of the object and indirectly to georeference the results of individual variants (Table 3).

**Figure 2:**
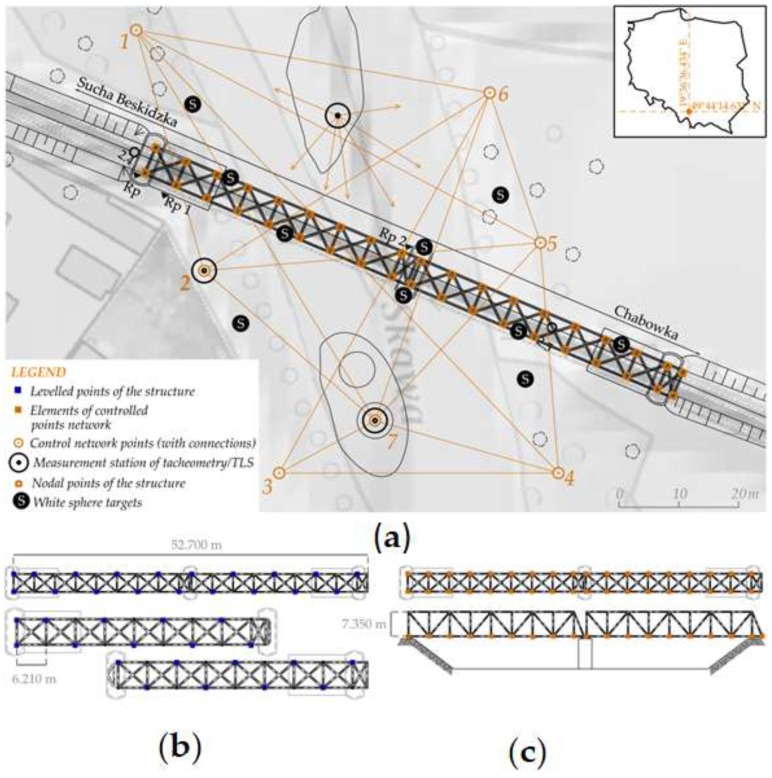
Measurement situations; (**a**) Control network; (**b**) Leveled points of the structure, top view; (**c**) Elements of the controlled point network, top view, back view.

Variant I was a classic determination of structure’s mechanics, without the need for the design and marking of points. Protrusions of the main girders were measured by precise leveling; their horizontal location coincided with the nodal points of the structure and, therefore, with the controlled point network (Figure 2b). Variants (**II**) and (**III**) required design and point marking. These tasks concerned both the design and permanent marking of the control network as well as the network of controlled points (black and white targets, B&W targets) (Figure 2a,c). A Leica NA3003 (with invar staff Leica GPCL2) by Leica Geosystems, Trimble VX, and Z+F Imager 5010 were used in every series of period measurements (Table 4). The measurement procedure assumed that variants (**II**) and (**III**) were made at the same real time, alternately, from the same measurement stations (Figure 2a).

The control network has been designed according to the PKP PLK S.A. regulations (Figure 2a). As a result, a control network established the global reliability indicator, which was close to 90%. The high relative reliability of the control network was supposed to reduce the RMS (Root Mean Square) of the registration of point clouds. Periodic tests of the stability of the control network, which preceded each periodic measurement of the bridge, indicated their situational and vertical stability.

The periodic measurements were carried out in three series: in May 2014 (first series), October 2014 (second series), and June 2015 (third series). The periods of measurement were a consequence of breaks in rolling stock traffic on railway line No. 098. The temporary diversion of rolling stock traffic away from the railway line was a consequence of upgrade operations on railway line numbers 097 and 098 Kraków–Zakopane. It was a part of the Multi-Year Railway Investment Programme, which was financed from the European Operational Infrastructure and Environment Programme. The upgrade activities were part of the process of introducing the high-speed rail to Poland. 

### 2.3. Procedures for Determining the Optimal Post-Processing of 3D Data

The endeavor to identify the most appropriate methodology of determining the stability of a railway bridge using TLS was an argument for determining the displacements of the object in different, specific case studies and using 3D data differentiated by preliminary processing. The vertical displacements of the railway bridge using TLS data were determined by identifying the spatial displacements of black and white targets of the controlled point network, by obtaining differential point cloud models, and also by comparing their generalizations–surface models (Table 5). The last two case studies were based on sets of input data for which different preliminary processing methods were used. 

The first set of input TLS data did not require preliminary processing (except manual filtration). The second and the third sets were the results of registration with a georeferenced control network. In addition, the data in the third set were filtered.

The registration process of point clouds was done in two steps (Table 6) using commercial Leica Cyclone software. The first step of the registration linked point clouds used the target-to-target method and the points of the control network as a home ScanWorld. The second step of the registration was the altitude correction of the point cloud from the first step. For this purpose, the results of the precise leveling of white reference spheres were used. During the measurement, the white spheres were located in the scanning space: on the abutments, the bridge, and the ground (Figure 2a). The spatial location of the spheres provided an appropriate correction of the height of the point cloud in the second step of the registration.

The three methodological approaches analyzed in the three case studies were not accidental. They are the most accessible and frequently used solutions. The periodic comparison of changes in the spatial position of B&W targets (case study 1) was the application of a popular total station scanning solution. The idea of case study 1 denies the complexity of point cloud analyses. However, a point analysis of the same objects using TLS and tacheometers provided a preliminary verification of the quality of the scan data. Girardeau-Montaut et al. [45] and Lague et al. [46] investigated the periodic displacements and deformations of point clouds by generating differential point cloud models (case study 2) (with the closest point approach). The results of these studies indicated that the cloud-to-cloud analyses were sufficient to indicate detection changes. Mill et al. [47] and Lõhmus et al. [35] offered a contrary opinion. They believed that the periodic comparison of TLS data using differential surface models (case study 3) reduced measurement noise, which is extremely valuable. Regular research on bridge displacement with the TLS technology requires one optimal methodology. However, one should reflect upon what support for TLS data from traditional measurements is necessary, and which post-processing steps are necessary. Next, we investigate what should be kept in this respect and what should be removed in the context of work optimization.

Case study 1 led to the determination of spatial displacements of elements of the controlled point network using the second set of input TLS data. The periodic coordinates of black and white targets were estimated using Leica Cyclone algorithms, and their accuracy was discussed. The accuracy of linear coordinates of every element of the controlled point network depends on the RMS of registration and RMS of fitting a regular object (white sphere) into a point cloud [48,49]. Next, the displacement of every single element of the controlled point network was calculated using commercial Geonet software (for series of measurement: first to second, first to third). The results of the 3D analysis were compared to their reference (Table 5) (displacements of the same elements of the controlled point network obtained with reflectorless tacheometry). 

Case study 2 was based on the determination of the vertical displacements of the main girders of the bridge by a comparison of point clouds from two periods (for the first, second, and third sets of data, each in the first to second and first to third series). The differentiation was performed in the CloudCompare software, using the nearest neighbor distance algorithm. In the process of generating the differential model of the point clouds, the maximum distance (0.032 m—the maximum design deflection of the bridge) was assumed, above which the values of the determined displacements would be considered as incorrectly identified. The a priori accuracy $\sigma {\left(dV\right)}^{TLS}$ (standard deviation of vertical displacements determined by TLS) has been determined (according to researchers from the Technical University of Tallinn [47]) for the estimated displacements of the protrusions of the main girders. The results of the 3D analysis were compared to their reference (Table 5) (the displacements of the protrusions of the main girders which were determined using precise leveling).

Case study 3 led to the determination of the vertical displacements of the main girders of the bridge as a result of a juxtaposition of two surface meshes following their generation in commercial Geomagic Wrap software. A comparative analysis was performed for the second and third sets of data in the first to second and first to third series in Geomagic Control software using the directional algorithm with vector (x, y, z) = (0, 0, 1). The resulting displacements of the nodes of the main girders were evaluated for accuracy (as described in case 2) and compared with their reference (Table 5).

A two-sample Kolmogorov–Smirnov test (K-S) was applied to every case study to compare two sets of displacements determined by TLS data (F_1_(TLS)) and their references (F_2_(REF)). The K-S test facilitated the juxtaposition of the empirical distribution functions of the first and the second sample, for which the sizes is lower than using the *t*-Student test. The K-S test is popular in civil engineering. It is a good statistic for testing the compatibility of displacements. It is sensitive to differences in both the location of the empirical cumulative distribution functions of two samples and also to their shapes [50]. To verify the compatibility of the displacement, the authors verified:

**Hypothesis** **H0.** Null hypothesis: H0: F_1_(TLS) = F_2_(REF): The empirical distribution functions of the first and the second sample respectively are identical.

versus

**Hypothesis** **H1.** Alternative hypothesis: H1: F1(TLS) ≠ F2(REF): The empirical distribution functions of the first and the second sample respectively are not identical.

The significance level (α) for the statistic was 1%. The cluster analyses of the first and the second sample were made with the cluster size equal to the accuracy of TLS registration (1 mm), according to the principle that the size of a single cluster should not be greater than the unit adopted for a defined scale problem [51]. 

In addition to the K-S test, which checked for the common population of displacements, for the results of the second and third case studies, the parameters of assessing the accuracy of the differences in displacement values ($\delta dV_{i}$) were calculated for every single node of the main girders (i), which were obtained by TLS ($dV_{i}^{TLS}$) and by precise leveling ($dV_{i}^{LEV}$):

(1) $$\delta dV_{i}=\;dV_{i}^{LEV}-\;dV_{i}^{TLS}$$

where:

$\delta dV_{i}$—differences in the displacement values of the single node (i) of the main girders,
$dV_{i}^{LEV}$—vertical displacements of the single node (i) of the main girders determined by leveling,
$dV_{i}^{TLS}$—vertical displacements of the single node (i) of the main girders determined by TLS.

The parameters for the assessment of the accuracy of the differences in displacement values $\delta dV_{i}$, which should facilitate the assessment and identification of the optimal methods for bridge stability investigation using TLS, included: the standard deviation (*s*), mean deviation (*D*), maximum deviation ($\delta_{max})$, and average deviation value $(\delta_{avg}$). The value of the maximum deviation would indicate the occurrence of outliers, while the value of the average deviation could indicate systematic errors between the two displacement measurement methods.

## 3. Results and Discussion

### 3.1. TLS Data Post-Processing

**Figure 3:**
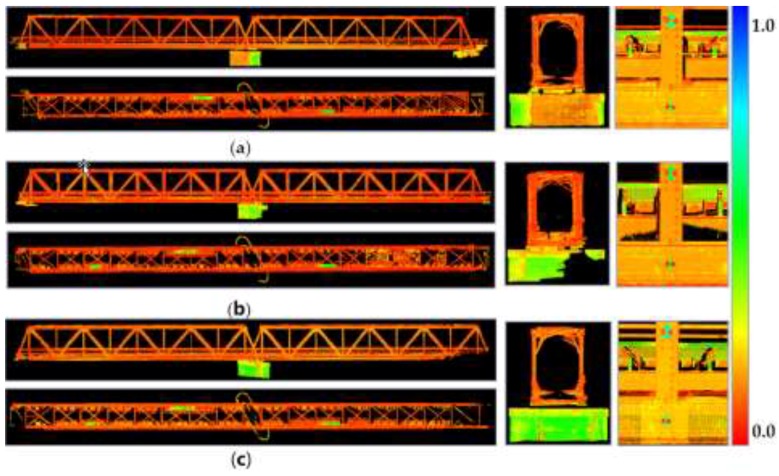
Point clouds of the bridge after registration by using the scale of intensity map (captured by Z + F 5010 and visualized in Leica Cyclone 9.2) in order: back view, top view, cross-section, detail; (**a**) first series; (**b**) second series (TLS data before static loading in [42]; (**c**) third series.

The two-step registration of point clouds resulted in 3D research material with registration accuracy not exceeding 0.001 m (MAE, mean absolute error) (Figure 3, Table 7). In structural stability studies, the 1-mm accuracy of registration is an absolute criterion for qualifying the usefulness of the TLS technology in displacement and deformation analysis [35].

The two-step registration of the point clouds facilitated constant control over the process by verifying the links between successive scan stations, while the correction of the cloud height improved the altitude quality of the TLS data, which is usually poorer than the situational one. The use of control network coordinates as registration bases (home ScanWorld) improved the registration geometry. The registration results confirmed the correctness of the design and marking of the control network, which was dedicated to TLS measurements. Should the configuration be incorrect, the geometry of the scan station control network system would cause an increase in the MAE and RMS values [48].

As a result of the filtration of the point clouds, the spatial data set has been reduced by nearly 11% (Table 7). The relatively constant level of noise reduction was a consequence of a relatively constant quality of periodic point clouds, which were acquired by the same measuring instrument and prepared by the same operator. The neighborhood algorithms (SOR (Statistical Outlier Removal) filter, noise filter) reduced the frequency of the measuring noise. A two-sided algorithm for slimming three-dimensional data (bilateral filter) smoothed 3D data for the railway bridge using the intensity values of the beam of reflection, and therefore sharpened its edges.

### 3.2. Case Study 1—Controlled Point Network Analysis

**Figure 4:**
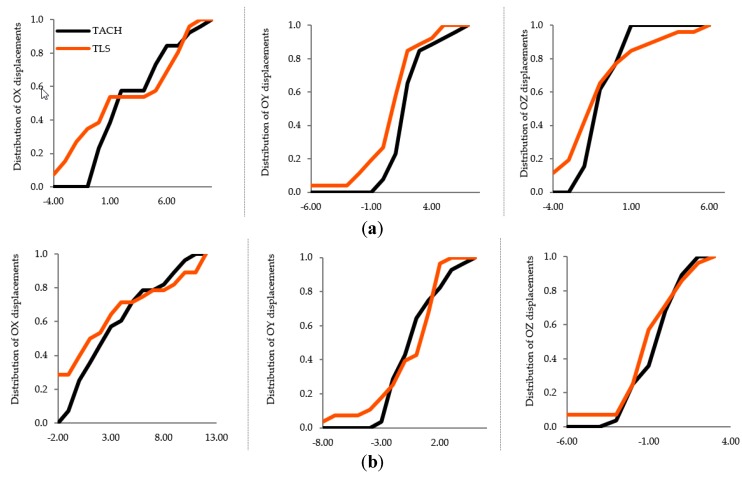
Distribution of spatial displacements TLS vs, TACH; *OX* axis: Clusters, *OY* axis: Distribution of displacements; (**a**) Epochs 1–2, (**b**) Epochs 1–3.

Verification of the compatibility of two samples of displacements (determined independently using two different technologies of reflectorless measurements) was performed for sets of horizontal displacements: on the *OX* axis of the system, on the *OY* axis of the system, and for sets of vertical displacements. The results of the two-sample K-S test (Table 8) confirmed the null hypothesis about the identity of distributions of random variables (for every set of displacements). These hypotheses facilitated the conclusion that the samples came from the same population. The statistical compatibility of the distribution in the two independent samples is illustrated by the diagram of the displacement distribution functions (Figure 4). 

The results of case study 1 show that the reflectorless tacheometry measurement (made in not less than two series) gives results similar to the fitting of clearly identifiable targets into a dense point cloud after registration. The correctness of the geometry of periodic point clouds of the bridge may be a consequence of the georeference, which requires design and marking as well as regular verification of the control network stability. 

Also, the monitoring of changes in the position of the black and white markers implies the need to prepare a research object. Case study 1 is a solution that meets the accuracy criteria for reflectorless measurements, but does not meet the conditions of a quick measurement (one that does not require preliminary work). It does not test the object globally, but it does test it point-wise. However, the demonstrated compliance of point displacements confirms the validity of using TLS data for displacement measurement.

### 3.3. Case Study 2—Point Clouds Analysis

**Figure 5:**
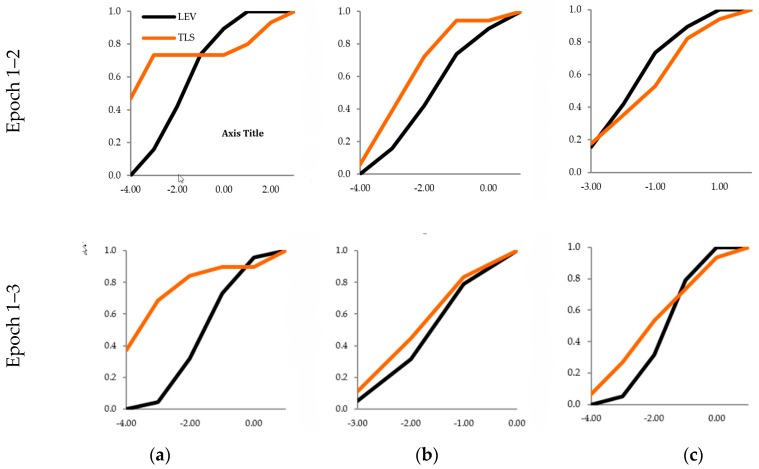
Distribution of vertical displacements TLS vs. LEV; *OX* axis: Clusters, *OY* axis: Distribution of displacements; (**a**) First set of data; (**b**) Second set of data; (**c**) Third set of data.

The two-sample K-S test for two sets of periodic data of the railway bridge girders (determined by precise leveling and a cloud-to-cloud analysis) showed that the samples belong to the same population only in the case of the second and third sets of input data (Table 9). The graphic compatibility of random variables is presented in an empirical distribution diagram (Figure 5).

The direct results of laser scanning (first sets of input data) significantly reduced the scope of research. Apart from terrestrial laser scanning, which was carried out with the highest possible resolution, the solution did not require a control network for georeferencing, precise measurements of the height of white target spheres, or any special preparation of the object for the measurement. The disadvantage of the solution investigated in this case study was the limited control over the results and the risk that the algorithm of point cloud registration (by fitting 3D data with a cloud-to-cloud algorithm—C2C) would lose significant information about displacements in the light of no control network. The values of the displacements determined in case study 2 for the first sets of input data were subjected to distortions at the stage of 3D data registration. This was due to the least squares method, which minimizes the differences between scans during registration.

The use of case study 2 for the first sets of input data might seem unjustified, but the literature contains such solutions. They are presented in the research work of a team of specialists led by Girardeau-Montaut, for example. The team sought to develop a cloud-to-cloud comparison algorithm that would quickly and reliably indicate structural displacements and deformations [50]. Displacement detection based on the comparison of periodic point clouds was also presented in a work on landslide monitoring in Castellfollit de la Roca [52].

### 3.4. Case Study 3—Surfaces Meshes Analysis

**Figure 6:**
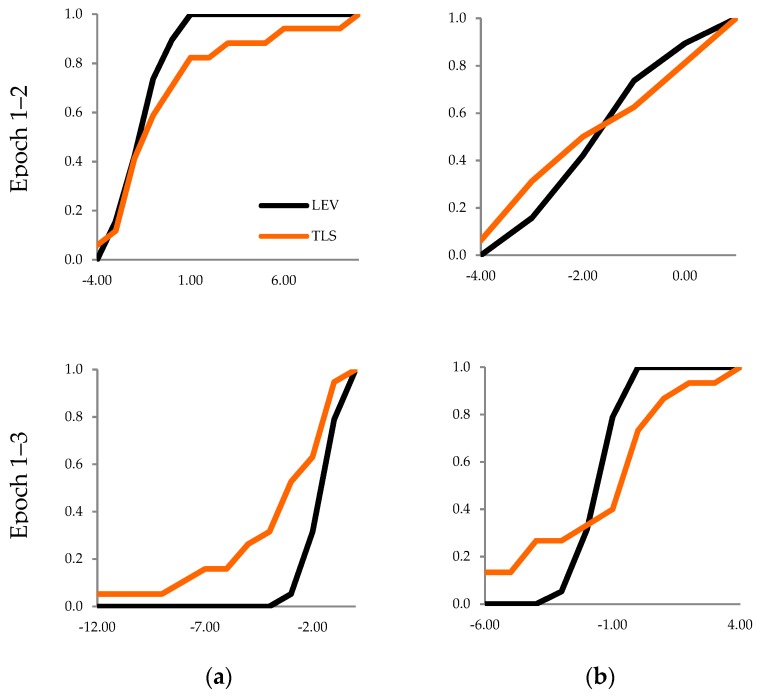
Distribution of vertical displacements TLS vs. LEV; *OX* axis: Clusters, *OY* axis: Distribution of displacements; (**a**) Second set of input data, (**b**) Third set of input data.

The two-sample K-S test for two sets of periodic vertical displacements, which were obtained by precise leveling and by comparing the surface meshes of girders, showed the identical empirical distribution functions for the first and the second sample (Table 10, Figure 6). A common population was identified for both surface meshes generated based on the second sets of input data and surface meshes from the third sets.

When point clouds and mesh surfaces were compared, it was found that the a priori accuracy of vertical displacement of every single protrusion of girder $\sigma {\left(dV\right)}^{TLS}$ is equal to ±2.4 mm. The parameters for assessing the accuracy of differences of displacement values ($\delta dV_{i}$) were calculated for every single solution in case studies 2 and 3. The average values of the parameters facilitated a comparison of the effectiveness of case studies in the context of the expected accuracy (Table 11).

The results of case study 2 showed that the registered point clouds of the object with a georeferenced control network (second set of input data) led to the determination of vertical displacements of the railway bridge that were the most similar to the ones obtained by precise leveling (Table 11). A comparison of the two periodic point clouds (case study 2, the first set of input data) showed that a huge amount of point cloud data is not enough to study the vertical displacements of structures with high certainty. 

The filtration algorithms in the comparative analysis of point clouds do not ensure the greater compatibility of displacements determined using the TLS technology with the results of traditional land surveying measurements. However, the filtration process contributes significantly to the reduction of systematic errors ($\delta_{avg}$), and as a consequence, leads to an increase in the symmetry of the scatter results around the mean in the analysis of the histogram of 3D data. However, the decisive reducer of error is the use of coordinate points of the control network in the registration process, which are determined with high reliability and with the use of precise land surveying methods. The case study 3 results showed a higher compliance with the reference measurement results for surface meshes that were generated on the basis of the second sets of input data with the use of the filtration algorithms (Table 11). 

Case study 3 did not identify any vertical displacements for the edge protrusions of the girders. As a result, the use of a differential point cloud model in the stability study is only possible for point clouds with a density of at least 2 mm. In the case of a lower resolution, the numerical data of the object are lost in the process of the generation of surface meshes and in the process of the filtration and generation of surface meshes. In the literature, the differentiation of surface models is a suitable solution for surface structures such as tunnels [53] or water dams [54]. The use of surface meshes is recommended for numerical data with a high degree of resolution and after filtration, which increases the degree of reliability of 3D data and almost completely eliminates systematic errors.

The results of epochal TLS data analyses (Table 11) are in relative agreement with the results of bridge stability research during static load tests [42]. The differences only apply to case study 3 for the third set of data. In the case of tests during static load tests [42], a significant reduction of the assumed $\sigma {\left(dV\right)}^{TLS}$ to the value of ±1.3 mm was obtained. All the errors of epochal measurements (centering, leveling), changing external conditions of the measurements (measurements at different times of the year), as well as georeferences of epochal point clouds based on the control network coordinates after the epochal stability tests did not significantly impact the results of differentiation of epochal 3D data. The methodical compliance of epochal results and during static load tests results [42] indicates the full reliability of TLS data in the study of vertical displacements of bridges.

## 4. Conclusions

The epoch study of the stability of railway bridges using terrestrial laser scanning has demonstrated that a strictly defined methodology of measuring and processing TLS data in synergy with traditional land surveying measurements facilitates a reliable determination of the epochal vertical displacements of whole bridges. 

The optimum method for investigating the stability of a bridge using terrestrial laser scanning, mainly due to its accuracy and comprehensive nature of the dislocation, consists in the differentiation of high-resolution point clouds supported by the georeferencing of a precisely determined control network with high relative reliability. The recommended processing methods indicate the necessity to provide a sound control network georeferencing for an object’s spatial data. In this regard, the conclusions of the studies are consistent with those obtained during static load tests [42]. Georeferencing requires a control network, which entails a design, marking, and in the case of epochal measurements, periodic measurements. A certain advantage of the methods is the comprehensive analysis of the changes within the structure, which does not require design and installation operations regarding controlled points.

As was the case for static load displacements [42], filtration algorithms that improve the reliability of spatial data do not ensure a better consistence of displacement values determined using TLS with the results of traditional approaches in methods employing the differentiation of point clouds in georeferenced control networks. What is essential as regards the measurements of the whole object is that epoch data filtration resulted in a significant reduction of cloud points at the ends of bridge spans, which resulted in no data regarding the displacement of those elements.

The optimum methods for processing TLS data offered a ±1 mm accuracy of the determination of the structure’s mechanics. It is a poor value compared to precise surveying methods, but when applied for a selective and quick assessment of railway infrastructure regarding its construction, upgrading, or restoration, it is a justified and comprehensive alternative solution for epochal measurements and also during static load tests [42].

## Figures and Tables

**Table 1:** Conflicts between PKN (the Polish Committee for Standardization) standards and PKP PLK S.A. (the Polish national railway) regulations in terms of load tests, based on [16].

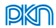	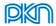	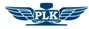	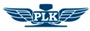	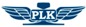
PN-89/S-10050	PN-99/S-10040	Id-16 Instructions (2005)	Id-16 Instructions (2014)	PKP Standards (2009)
**Static Load Test**				
L * > 21 m	Every bridge	Every bridge	Not applicable	Every bridge
**Dynamic Load Test**				
L > 21 m	L = approx. 15 m	Steel: L > 21 m Concrete: L > 10 m	Not applicable	L > 21 m

* L—the length of the bridge.

**Table 2:** Variants of the object displacement measurements. TLS: terrestrial laser scanner.

		Goals
I	Precise leveling of bridge spans	The vertical displacements of protrusions of the main girders of the bridge
		Reference measurements for TLS results
II	Reflectorless precision tacheometry of controlled points	The displacements of controlled point network
		Reference measurements for TLS results
III	Terrestrial laser scanning	The displacements of the bridge determined using: - TLS data of the controlled point network - processing of point clouds - processing of surface meshes

**Table 3:** Measurement methods and their aims.

	Aims
Precise leveling	Periodic precise leveling of the control network
	Precise leveling of white sphere targets
	Periodic precise leveling of the main girders of the bridge
Precise tacheometry, reflection-based measurement	Periodic measurement of the control network
Precise tacheometry, reflectorless measurement	Periodic measurement of the controlled point network
Terrestrial laser scanning	Periodic measurement of the bridge

**Table 4:** The technical characteristics of measuring instruments (based on [44]).

Instrument	Technical Parameters
Leica NA3003	Measuring distance: 1.3–100 m
	Precision of distance measurement: ± (3 mm + 5 pmm)
	Accuracy per 1 km of double leveling: 0.4 mm (invar staff), 1.2 mm (standard staff)
Trimble VX	Maximum measuring distance: 250 m
	Precision of distance measurement: ± (1 mm + 2 pmm)
	Angular accuracy 1″
Z+F Imager 5010	Maximum measuring distance: 187 m
	Beam divergence < mrad (full angle)
	Beam diameter approx. 3.5 mm per 1 m
	More than 1 million pixel/sec maximum measurement rate

**Table 5:** Procedures of post-processing of TLS data, defined in three case studies.

	CASE STUDY 1 Controlled Point Network	CASE STUDY 2 Point Clouds	CASE STUDY 3 Surface Mesh
Methodology of determining displacements by TLS	periodic comparison of changes in the spatial position of elements of the controlled point network	periodic comparison of point clouds by generating differential point cloud models of the main bridge girders, analysis of vertical displacements	periodic comparison of surface models by generating differential surface models of the main bridge girders, analysis of vertical displacements
Reference measurements	displacements of elements of the controlled point network, determined by reflectorless tacheometry	the vertical displacements of nodes of the main girders, determined by precise leveling	the vertical displacements of the nodes of the main girders, determined by precise leveling
	-	point clouds of the object	-
Sets of input TLS data	registered point clouds of the object, with the georeferenced control network	registered point clouds of the object, with the georeferenced control network	registered point clouds of the object, with the georeferenced control network
	-	second set of input data with the use of filtration algorithms	second set of input data with the use of filtration algorithms

Based on [42,46].

**Table 6:** Diagram of the point cloud registration steps.

**Stage ^1^**	**Tie Points**		**Georeference Data**	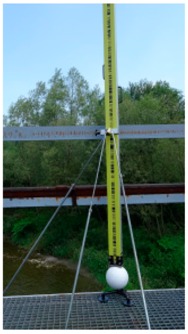
1	HDS (High-Definition Surveying) targets	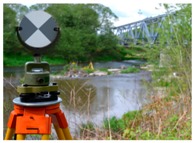	(X, Y) of points of the control network	
2	white sphere targets ^2^	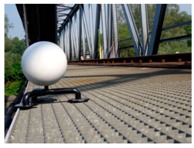	(X, Y) of sphere targets from the results of the first stage (H) of the centers of the sphere targets from precise leveling	

^1^ Based on [46]. ^2^ Device for stabilizing reference spheres on terrain surface protected by registered utility model No. W.126075, creator: Maria Makuch.

**Table 7:** Results of TLS data and TLS data filtration. MAE: mean absolute error.

**REGISTRATION**		**First Series**	**Second Series**	**Third Series**
MAE [m]		0.001	0.001	0.001
RMS [m]		0.002	0.001	0.002
**FILTRATION**		**Number of Elements in Point Clouds**		
**Filtration Stage**	**Filtration Algorithm**	**First Series**	**Second Series**	**Third Series**
-	Periodic point cloud	48,531,393	53,926,203	24,984,892
1	SOR filter	45,665,543	49,787,683	24,368,852
2	Noise filter	43,137,503	47,036,862	22,828,872
3	Bilateral filter	43,053,399	47,020,857	22,828,515
Percentage of the reduction of the elements of point clouds [%]		11	13	9

**Table 8:** Two-sample Kolmogorov–Smirnov (K-S) test results; TLS vs. TACH (tacheometry), case study 1; (**a**) Epochs 1–2; (**b**) Epochs 1–3.

**(a) Epochs 1–2**				
**Displacement**	**Significance Level α**	**Cluster Analysis**	**Statistic Value λ (D = sup| F_1_(TLS) − F_2_(TACH)|)**	**Resultant Hypothesis**
along the *OX* axis	α = 0.01	0.001 m	λ = 1.25 < λ_α_ = 1.63	H_0_
along the *OY* axis			λ = 1.25 < λ_α_ = 1.63	H_0_
vertical			λ = 0.97 < λ_α_ = 1.63	H_0_
**(b) Epochs 1–3**				
**Displacement**	**Significance Level α**	**Cluster Analysis**	**Statistic Value λ (D = sup| F_1_(TLS) − F_2_(TACH)|)**	**Resultant Hypothesis**
along the *OX* axis	α = 0.01	0.001 m	λ = 1.07 < λ_α_ = 1.63	H_0_
along the *OY* axis			λ = 0.80 < λ_α_ = 1.63	H_0_
vertical			λ = 0.80 < λ_α_ = 1.63	H_0_

**Table 9:** Two-sample K-S test results TLS vs. LEV (leveling), case study 2; (**a**) First set of data, (**b**) Second set of data, and (**c**) Third set of data.

**(a) First Sets of Data**				
**Epoch**	**Significance Level α**	**Cluster Analysis**	**Statistic Value λ (D = sup| F1(TLS) − F2(LEV)|)**	**Resultant Hypothesis**
1–2	α = 0.01	0.001 m	λ = 1.66 > λα = 1.63	H1
1–3			λ = 2.04 > λα = 1.63	H1
**(b) Second Sets of Data**				
**Epoch**	**Significance Level α**	**Cluster Analysis**	**Statistic Value λ (D = sup| F1(TLS) − F2(LEV)|)**	**Resultant Hypothesis**
1–2	α = 0.01	0.001 m	λ = 0.92 < λα = 1.63	H0
1–3			λ = 0.82 < λα = 1.63	H0
**(c) Third Sets of Data**				
**Epoch**	**Significance Level α**	**Cluster Analysis**	**Statistic Value λ (D = sup| F1(TLS) − F2(LEV)|)**	**Resultant Hypothesis**
1–2	α = 0.01	0.001 m	λ = 0.62 < λα = 1.63	H0
1–3			λ = 0.63 < λα = 1.63	H0

**Table 10:** Two-sample K-S test results TLS vs. TACH, Case study 3; (**a**) Second set of data; (**b**) Third set of data.

**(a) Second sets of data**				
**Epoch**	**Significance Level α**	**Cluster Analysis**	**Statistic Value λ (D = sup| F_1_(TLS) − F_2_(LEV)|)**	**Resultant Hypothesis**
1–2	α = 0.01	0.001 m	λ = 0.56 < λ_α_ = 1.63	H_0_
1–3			λ = 1.45 < λ_α_ = 1.63	H_0_
**(b) Third sets of data**				
**Epoch**	**Significance Level α**	**Cluster Analysis**	**Statistic Value** **Λ (D = sup| F_1_(TLS) − F_2_(LEV)|)**	**Resultant Hypothesis**
1–2	α = 0.01	0.001 m	λ = 0.46 < λ_α_ = 1.63	H_0_
1–3			λ = 0.82 < λ_α_ = 1.63	H_0_

**Table 11:** Parameters of assessing the accuracy of the differences in displacement values ($\delta dV_{i}$); (**a**) Case study 2; (**b**) Case study 3.

**(a) Case Study 2**	**First Set of Data**	**Second Set of Data**	**Third Set of Data**
$\sigma {\left(dV\right)}^{TLS}$ *	±2.4	±2.4	±2.4
s *	±2.3	±1.2	±1.5
D *	±1.9	±0.9	±1.0
$\delta_{max}$ *	±4.0	±2.5	±3.2
$\delta_{avg}$ *	0.8	0.5	-0.2
**(b) Case Study 3**		**Second Set of Data**	**Third Set of Data**
$\sigma {\left(dV\right)}^{TLS}$ *		±2.4	±2.4
s *		±4.3	±2.2
D *		±2.5	±1.3
$\delta_{max}$ *		±12.9	±4.5
$\delta_{avg}$ *		1.7	0.1

* [mm].
